# Integrating multidisciplinary perspectives in complex anal fistula management: a blueprint for future research and precision surgery

**DOI:** 10.1097/JS9.0000000000000981

**Published:** 2023-12-06

**Authors:** Jin Xu, Zubing Mei, Qingming Wang

**Affiliations:** a Department of Anorectal Surgery, Baoshan District Hospital of Integrated Traditional Chinese and Western Medicine; b Department of Anorectal Surgery, Shuguang Hospital, Shanghai University of Traditional Chinese Medicine; c Anorectal Disease Institute of Shuguang Hospital, Shanghai, China

*Dear Editor,*

Anal fistula is a common anorectal disease, with an annual prevalence of 8.6–10 cases per 10 000 population^1^. Anal fistulas can be classified as ‘simple’ or ‘complex’ based on their complexity. Simple anal fistulas encompass intersphincteric and low transsphincteric fistulas that engage less than 30% of the external sphincter. Fistulas that are challenging to manage, have a high risk of recurrence, and a potential of disturbing continence following surgical intervention are deemed complex. This includes types such as transsphincteric fistulas (those that involve greater than 30% of the external sphincter), suprasphincteric, extrasphincteric, horseshoe fistulas, anterior fistulas in women, or anal fistulas associated with inflammatory bowel disease (IBD). Recurrent or branching fistulas are also categorized as complex^2,3^. The primary means of definitively treating anal fistula is surgical intervention. The overarching goals of surgical treatment for fistula-in-ano are the assurance of a cure, reducing the postoperative recurrence rate, and preserving anal function to the greatest extent possible^4^.

Fistulotomy is a universally accepted and standard surgical procedure for treating simple anal fistulas^5,6^. This procedure boasts a high cure rate, inclusive of thorough obliteration of the internal opening and associated epithelialized tracts. However, the aggressive laying-open technique can inflict inevitable damage to the anal sphincter, resulting in varying degrees of postoperative incontinence. More complex fistulas, involving a significant portion of the anal sphincter, threaten more substantial damage to the anal sphincter, potentially leading to poor functional outcomes. Due to this inherent risk, multiple sphincter-preserving treatments for complex anal fistulas, such as ligation of intersphincteric fistula tract (LIFT)^4^, endoanal advancement flap repair (EAFR)^7^, video-assisted anal fistula treatment (VAAFT)^8^, and anal fistula plug (AFP)^9^ techniques, have been advanced in recent decades. However, their long-term performance often leaves much to be desired.

While the sphincter-preserving treatments can remedy specific types of anal fistulas without significantly compromising anal function, there is a dearth of high-quality evidence to champion an optimal procedure for particular types of anal fistulas due to inconsistent clinical efficacy^10^. For instance, the reported postoperative recurrence rate of anal fistulas treated by the LIFT procedure can climb up to as much as 60%^11^. Recurrence of anal fistula is a frequent and potentially debilitating consequence after anal fistula surgery^12^. Numerous factors influence the evolution and outcomes of patients with anal fistulas. Mei *et al.* ^12^. conducted a meta-analysis to collate and vet the credibility of evidence of potential risk factors for anal fistula recurrence after surgery. Their analysis demonstrated significant increases in the risk of postoperative recurrence rate with history of previous anal surgery, high transsphincteric fistula, undetected internal opening, and presence of horseshoe extensions, seton placement surgery, and multiple fistula tracts. A consensus was reached^13^ from an evidence-based Delphi consultation survey on 14 statements on the risk factors for anal fistula recurrence across three domains including patient-related risk factors (comorbid colitis, inflammatory bowel disease, and use of immunosuppressants), fistula-related factors (transsphincteric fistula, number of fistulas, horseshoe extension, undetected internal opening, location of anal fistula, recurrent fistula, suprasphincteric fistula, and height of the internal opening) and surgery-related factors (type of surgery, previous fistula surgery, and surgeon’s experience).

To date, no single surgical technique can claim the ‘gold standard’ status for the treatment of complex anal fistulas, leaving the management of such fistulas a formidable challenge for surgeons^6^. The difficulty in surgically treating complex anal fistulas chiefly stems from: (1) locating and treating the internal openings of certain anal fistulas with precision; (2) addressing the dichotomy between effective drainage and wound size reduction; (3) reconciling complete wound debridement with the preservation of anal function; and (4) resolving the contradiction between sphincter preservation surgery and long-term effects^10^.

An *et al.* ^14^ conducted a systematic review and network meta-analysis to evaluate the efficacy and safety of 13 surgical techniques for non-Crohn’s complex anal fistula (CAF). Analyzing data from 28 RCTs involving 2274 patients, the study found no significant differences in cure or recurrence rates across techniques. Nevertheless, fistulectomy might present a lower complication rate as compared to other methods. While the analysis of operating time, pain, and incontinence lacks closed loops, preliminary rankings suggest fistulectomy also may offer shorter operating times, with other methods like VAMLIFT and LIFT proposing lower postoperative pain and incontinence, respectively. Thus, fistulectomy might emerge as a superior option for CAF treatment.

While the article by An *et al.* ^12^ provides comprehensive insights into the comparative outcomes of surgical interventions for complex anal fistulas, certain limitations inherent in its design and findings need to be addressed:

Firstly, the meta-analysis integrates data from numerous randomized controlled trials (RCTs) that might differ significantly in their design, execution, and quality. Variations may arise in terms of patient selection criteria, surgical expertise, and postoperative care protocols, introducing systematic variability and potentially affecting the comparability of results. Secondly, the RCTs included in the meta-analysis are confined to non-Crohn’s complex anal fistulas (CAF), potentially limiting the generalizability of the findings. The complexity and pathophysiology of CAFs associated with Crohn’s disease might differ, thus necessitating cautious application of the conclusions to the broader CAF patient population. Thirdly, the absence of statistically significant differences in cure and recurrence rates among the surgical techniques does not preclude the possibility of clinically relevant differences that the study was not powered to detect. The operational definition of “cure” and the time frame for assessing recurrence could affect the interpretation of these outcomes. Fourthly, the network meta-analysis lacks closed loops for certain outcomes, such as operating time, postoperative pain, and incontinence. This omission precludes a more robust analysis that incorporates direct and indirect comparisons, refining the precision of the effectiveness rankings. Finally, the postoperative outcomes were measured at fixed time points—pain on day 1 and incontinence in month 1. These assessments fail to account for the dynamic nature of postoperative recovery and may not reflect longer-term functional outcomes or quality of life.

Despite its limitations, An and colleagues’ meta-analysis has several methodological strengths that bolster its scientific rigour. An expansive and thorough search of global databases ensures broad capture of available studies, reducing publication bias risk. Adhering to the gold standard for evaluating healthcare interventions, the study exclusively includes randomized controlled trials, enhancing validity by mitigating confounding and selection bias. The study design, incorporating the PICO (Population, Intervention, Comparison, Outcome) framework, lends clarity and precision to the research, making it clinically relevant. Sophisticated statistical tools like STATA and Review Manager are used in conjunction with network meta-analysis techniques, providing nuanced assessments of different treatments. The implementation of Surface Under the Cumulative Ranking curves (SUCRA) adds interpretability to treatment efficacy rankings. A multi-dimensional assessment evaluates various clinically relevant endpoints, fostering a comprehensive understanding of surgical interventions’ impacts. Collectively, these strengths validate the study’s contributions to existing literature and offer a solid foundation for informed decision-making in non-Crohn’s complex anal fistula management.

The clinical value of the systematic review and meta-analysis on complex anal fistula treatment is underscored by its potential to inform evidence-based practices and guide development. Future investigations stemming from this work should leverage rigorous randomized controlled trial designs with stratification to mitigate confounding factors and enhance the power to detect differences in treatment efficacy. Additionally, the combination of multidisciplinary approaches with novel biologic therapies warrants exploration. Studies should also endeavour to standardize outcome measures, incorporating both clinician-assessed and patient-reported metrics to provide a holistic view of therapeutic impact. The design of subsequent studies could benefit greatly from adaptive trial frameworks that permit modifications based on interim results, enhancing efficiency and ethicality by reducing patient exposure to inferior treatments. Furthermore, by embracing the principles of personalized medicine and investigating the role of genetic, microbial, and immunological factors in patient responses to treatment, the way could be paved for tailored therapeutic strategies.

The use of these future perspectives in study design has the potential to not only refine current treatment paradigms but also contribute to a more nuanced understanding of complex anal fistula pathophysiology, ultimately guiding clinicians towards more precise and patient-tailored management strategies.

## Ethical approval

This article does not require any human/animal subjects to acquire such approval.

## Sources of funding

This study is sponsored by Shanghai Sailing Program (21YF1433300), 2023 Shanghai Science and Technology Innovation Action Plan-Medical Innovation Research Project (23Y11921800) and “14th Five-Year Plan” Traditional Chinese Medicine Specialty and Traditional Chinese Medicine Emergency Capacity Improvement Project (ZYTSZK1-8).

## Author contribution

J.X.: conceptualization, study concept and design, drafting of the manuscript; Q.W.: drafting and validation of the manuscript. Z.M.: conceptualization, critical revision of the manuscript for important intellectual content. All authors critically reviewed and approved the final version of the manuscript before submission.

## Conflicts of interest disclosure

There are no conflicts of interest.

## Research registration Unique Identifying number (UIN)

1. Name of the registry: NA.
2. Unique Identifying number or registration ID: NA.
3. Hyperlink to your specific registration (must be publicly accessible and will be checked): NA.

## Guarantors

Zubing Mei, Jin Xu.

## Data availability statement

This correspondence did not yield data and thus no data were available.
